# Incarcerated hiatal hernia with afferent loop syndrome after laparoscopic total gastrectomy with Roux-en-Y reconstruction: A rare case report

**DOI:** 10.1097/MD.0000000000042525

**Published:** 2025-05-30

**Authors:** Hyun Wook Shin, Ki Bum Park

**Affiliations:** a Department of Surgery, Kyungpook National University Chilgok Hospital, Daegu, Republic of Korea; b Department of Surgery, School of Medicine, Kyungpook National University, Daegu, Republic of Korea.

**Keywords:** afferent loop syndrome, gastrectomy, hiatal hernia

## Abstract

**Rationale:**

Hiatal hernia is one of the rarest types of internal herniation that can occur after gastrectomy. We present a case of hiatal hernia accompanied with afferent loop syndrome.

**Patient concerns:**

An 82-year-old male, who had undergone laparoscopic total gastrectomy and D2 lymphadenectomy with Roux-en-Y reconstruction 10 months prior was admitted to the emergency room with acute abdominal pain.

**Diagnoses:**

A computed tomography revealed a hiatal hernia containing efferent and left-sided small bowel loops, dilation of the afferent limb, and dilation of the extrahepatic biliary duct due to afferent limb obstruction.

**Interventions:**

Laparoscopic reduction was significantly hindered by bowel edema. To ease the reduction of the small bowel, an incision was made to the diaphragm to ensure a bigger hiatal opening. After the reduction was complete, the hiatal opening was sutured to reduce the size of the diaphragmatic hiatus.

**Outcomes:**

Reduced small bowels were in viable condition. The patient was discharged eleven days after surgery without any complications.

**Lessons:**

The case demonstrates that hiatal hernia with afferent loop syndrome can occur after total gastrectomy with Roux-en-Y reconstruction. And, in such cases, incision of the diaphragm may be necessary for a safer bowel reduction.

## 1. Introduction

Internal hernia is a rare complication after gastrectomy that can lead to severe consequences. It is reported that internal hernia can occur in 0.2% to 1.7% of patients after undergoing gastrectomy for gastric cancer. The most common sites for internal hernia are jejunojejunostomy mesenteric defect and Petersen’s space accounting for 88% to 100% of internal hernia sites.^[1–3]^ Hiatal hernia, a distinct type of internal hernia, is exceptionally rare, with its rate of occurrence being 0.01% to 0.05% after gastrectomy.^[1,4]^

The use of laparoscopy for gastrectomy is increasing throughout the world due to its minimally invasive nature and reduced postoperative adhesions.^[2,5]^ However, the reduced formation of adhesions is a key factor that makes laparoscopic surgery one of the major risk factors for internal hernia. Additional risk factors include non-closure of mesenteric defects and low body mass index.^[2,3]^ Closure of mesenteric defects significantly decreases the chance of internal herniation.^[6]^

In this case report, we present a case of hiatal hernia accompanied with afferent loop syndrome after laparoscopic total gastrectomy with Roux-en-Y reconstruction.

## 2. Case report

An 82-year-old male presented to the emergency department with acute abdominal pain. Ten months earlier, he had undergone laparoscopic total gastrectomy and D2 lymphadenectomy with Roux-en-Y reconstruction due to gastric cancer. His initial surgery was completed without immediate complications and was discharged on postoperative day 7 in stable condition. There were no specific complaints at his 3- and 6-month regular follow-up, and abdomen–pelvis computed tomography on his 6-month follow-up showed no evidence of tumor recurrence or internal herniation.

**Figure 1.**
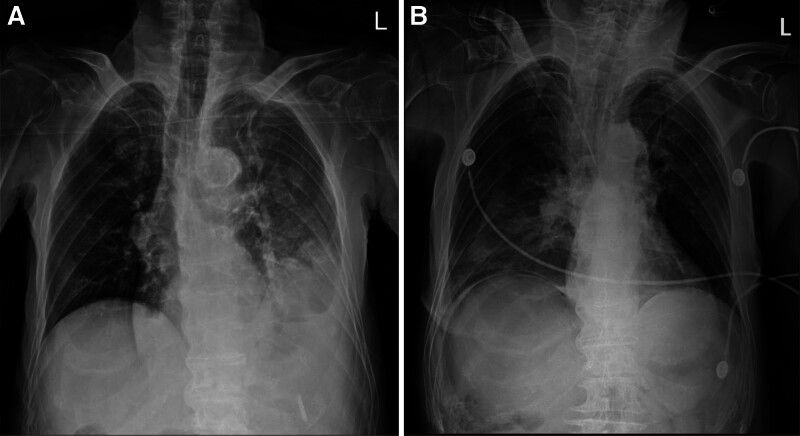
(A) Chest X-ray revealing a bowel shadow in the left thoracic cavity, indicating hiatal hernia. (B) Resolution of the lesion immediately after surgery.

**Figure 2.**
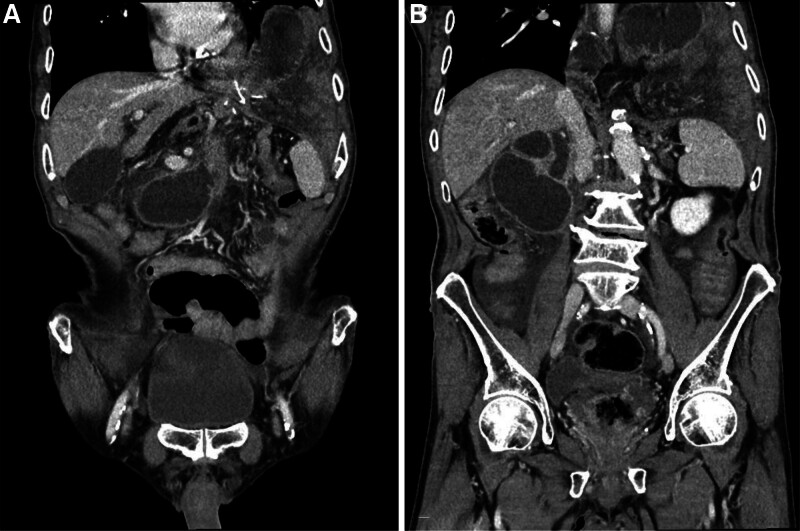
Abdomen–pelvis computed tomography before surgery revealing: (A) Distension of the afferent loop and hiatal hernia. (B) Distension of the gallbladder and common bile duct.

A chest X-ray taken in the emergency department displayed bowel shadow at the left thoracic cavity, indicating a hiatal hernia (Fig. 1A). An abdomen–pelvis computed tomography was performed, which confirmed a hiatal hernia containing efferent and left-sided small bowel loops, along with dilation of the afferent limb, and extrahepatic biliary duct due to afferent limb obstruction (Fig. 2). An emergency operation was undertaken for the reduction of the hiatal hernia.

**Figure 3.**
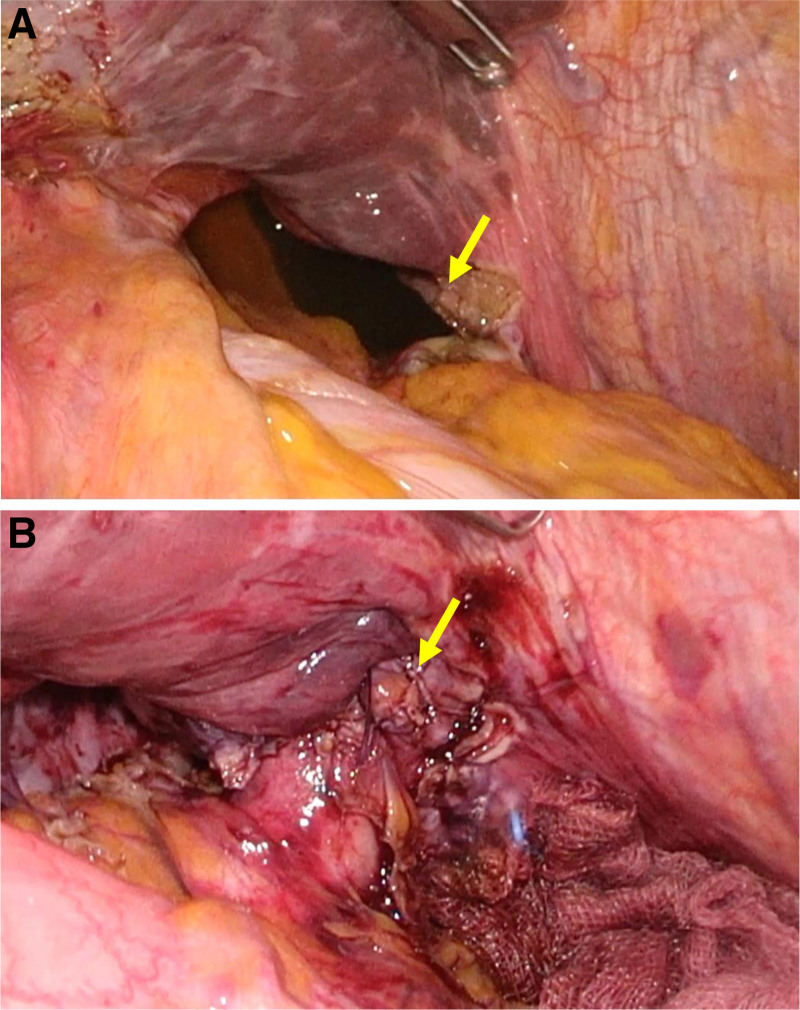
(A) Hiatal opening with partial transection of the diaphragm for successful reduction of the herniated bowel. (B) The size of the hiatal defect was reduced by suturing the transected diaphragm.

Distension of the afferent limb was noted, and the small bowel was herniated through the hiatus, with the jejunojejunostomy obstructing the hiatal opening. Laparoscopic reduction without additional incision to the diaphragm was initially attempted. However, due to edema of the small bowel, the reduction was unsuccessful. A partial transection of the diaphragm was performed to widen the esophageal hiatus (Fig. 3A). The herniated small bowel, including the jejunojejunostomy, was successfully returned to the abdominal cavity. The herniated small bowel showed no signs of ischemic changes and was in a viable condition. The transected diaphragm was sutured to reduce the size of the hiatal defect (Fig. 3B).

**Figure 4.**
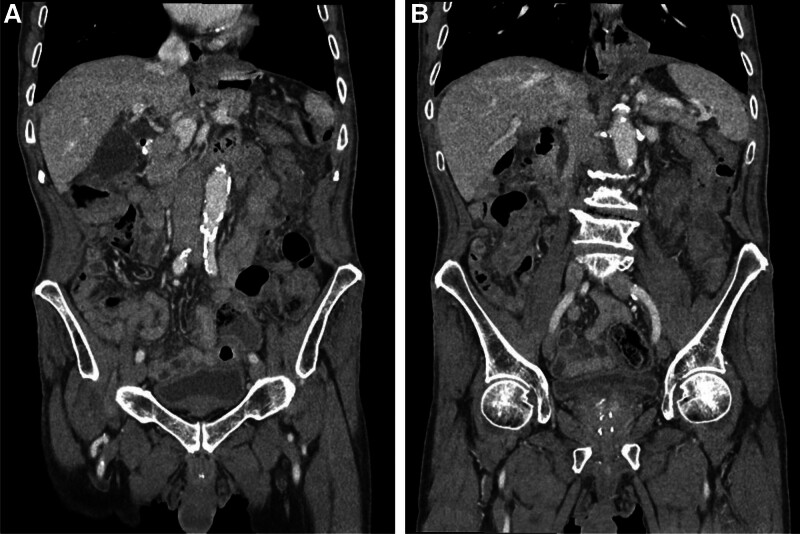
Abdomen–pelvis computed tomography after surgery revealing: (A) Resolved hiatal hernia and decompression of the afferent loop. (B) Mild dilation of extrahepatic bile duct.

After the surgery, the patient was admitted to the intensive care unit for 2 days. A postoperative chest X-ray revealed the resolution of the bowel shadow, indicating a successful reduction of the hiatal hernia (Fig. 1B). An abdomen–pelvis computed tomography scan performed 6 days after surgery revealed a resolved hiatal hernia with decompressed afferent and efferent limbs, and a mild dilation of the extrahepatic biliary duct (Fig. 4). The patient was allowed to resume a soft diet on postoperative day 7 and was discharged on postoperative day eleven without any complications.

## 3. Discussion

Although rare, internal hernia is a critical complication following gastrectomy. Hiatal hernia is one of the rarest types of internal hernia. A multi-institutional cohort study on the incidence of internal hernia after gastrectomy was conducted by Hiromichi et al. The study constituted of 8983 patients who underwent gastrectomy, among whom thirteen patients required surgical treatment for internal hernia. Of these thirteen patients, only 1 patient was presented with hiatal hernia (0.01%).^[1]^ Similarly, a single-center retrospective study by Ojima et al, which included 1943 patients who underwent surgery for gastric cancer, reported only 1 case of hiatal hernia (0.05%).^[4]^

The use of laparoscopy in gastric surgery is increasing worldwide. Laparoscopic surgery is minimally invasive and reduces the risk of postoperative adhesions, enabling earlier recovery of bowel motility and reduced hospital stay.^[2,5]^ However, studies have shown that the laparoscopic approach itself is a statistically significant variable for internal hernia with odds ratio ranging from 1.65 to 4.95 compared to the open approach. All authors stated that the reduction of postoperative adhesion allows for greater bowel mobility, which increases the likelihood of bowel herniating into mesenteric defects.^[2–4]^

Management of mesenteric defects during gastrectomy is controversial due to its technical difficulty and potential complications, such as hemorrhage, mesenteric hematoma, and ischemia of associated anastomosis.^[7]^ However, studies have shown that closure of mesenteric defect significantly reduces the incidence of internal hernia.^[2,8]^ Unlike mesenteric defects, the hiatal opening in total gastrectomy presents a unique situation, as the esophagojejunostomy is performed within the hiatal opening. Since the anastomosed esophagojejunostomy is larger than the esophagus alone, a slightly enlarged hiatal opening is often required. As a result, the hiatal opening is left unrepaired in most total gastrectomy cases. In the present case, the esophagojejunostomy slid upwards into the thoracic cavity, leaving the hiatal opening to be occupied only by the jejunum. This created sufficient space for the small bowel to herniate through. Therefore, in cases where the hiatal opening is larger than necessary, crus repair may be considered to reduce the risk of hiatal hernia.

Unlike other reported cases of hiatal hernia following total gastrectomy, this case is unique in that afferent loop syndrome occurred concurrently with the hiatal hernia. Since the jejunojejunostomy is bulkier than the small bowel alone, the small bowel distal to the jejunojejunostomy herniated through the hiatus, with the jejunojejunostomy blockading the hiatal opening. Thus, creating an obstruction of the afferent limb. Afferent loop syndrome is a rare complication that occurs in 0.2% to 1.0% of patients after gastrectomy with a Billroth II or Roux-en-Y reconstruction. This complication can result from various causes, including internal herniation, kinking at the anastomotic site, adhesions, stomal stenosis, a gastrointestinal stone, recurrent malignancy, and volvulus.^[9]^ It is important to note that hiatal hernia can also cause afferent loop syndrome. In such cases, surgical reduction is considered the treatment of choice.

Another critical point to consider while performing bowel reduction is the possibility of encountering bowel perforation, either preexisting or occurring during the procedure. Severe edema of the herniated bowel can develop since the mesentery is often herniated along with the bowel. In such cases, primary reduction can be challenging and may increase the risk of bowel injury. Therefore, a key technique for reducing edematous bowel in hiatal hernia is partial transection of the diaphragm to create a larger aperture. In the present case, the herniated bowel was successfully reduced without bowel injury by partially transecting the diaphragm.

## 4. Conclusion

Incarcerated hiatal hernia with afferent loop syndrome is an extremely rare but possible complication that can occur after laparoscopic total gastrectomy with Roux-en-Y reconstruction. For safe and successful bowel reduction during an incarcerated hiatal hernia, partial transection of the diaphragm should be considered. Additionally, to prevent hiatal hernia from occurring, crus repair should be performed if the hiatal opening is larger than necessary.

## Author contributions

**Conceptualization:** Ki Bum Park.

**Data curation:** Ki Bum Park.

**Formal analysis:** Hyun Wook Shin.

**Supervision:** Ki Bum Park.

**Writing – original draft:** Hyun Wook Shin.

**Writing – review & editing:** Hyun Wook Shin, Ki Bum Park.
